# Self-Assembly in Monoelaidin Aqueous Dispersions: Direct Vesicles to Cubosomes Transition

**DOI:** 10.1371/journal.pone.0003747

**Published:** 2008-11-18

**Authors:** Anan Yaghmur, Peter Laggner, Mats Almgren, Michael Rappolt

**Affiliations:** 1 Institute of Biophysics and Nanosystems Research (IBN), Austrian Academy of Sciences, Graz, Austria; 2 Department of Physical Chemistry, Uppsala University, Uppsala, Sweden; University of East Piedmont, Italy

## Abstract

**Background:**

In the present study, synchrotron small-angle X-ray scattering (SAXS) and Cryo-TEM were used to characterize the temperature-induced structural transitions of monoelaidin (ME) aqueous dispersion in the presence of the polymeric stabilizer F127. We prove that the direct transition from vesicles to cubosomes by heating this dispersion is possible. The obtained results were compared with the fully hydrated bulk ME phase.

**Methodology/Principal Findings:**

Our results indicate the formation of ME dispersion, which is less stable than that based on the congener monoolein (MO). In addition, the temperature-dependence behavior significantly differs from the fully hydrated bulk phase. SAXS findings indicate a direct L_α_-V_2_ internal transition in the dispersion. While the transition temperature is conserved in the dispersion, the formed cubosomes with internal Im3m symmetry clearly contain more water and this ordered interior is retained over a wider temperature range as compared to its fully hydrated bulk system. At 25°C, Cryo-TEM observations reveal the formation of most likely closely packed onion-like vesicles. Above the lamellar to non-lamellar phase transition at 65°C, flattened cubosomes with an internal nanostructure are observed. However, they have only arbitrary shapes and thus, their morphology is significantly different from that of the well-shaped analogous MO cubosome and hexosome particles.

**Conclusions/Significance:**

Our study reveals a direct liposomes-cubosomes transition in ME dispersion. The obtained results suggest that the polymeric stabilizer F127 especially plays a significant role in the membrane fusion processes. F127 incorporates in considerable amount into the internal nanostructure and leads to the formation of a highly swollen Im3m phase.

## Introduction

Curved membranes (non-lamellar structures) lay the basis for many new applications in controlling drug release from nanoparticulate systems [1]–[3] and as matrices for the entrapment and the crystallization of proteins [4]–[6]. The class of amphiphilic materials with the propensity to form non-lamellar nanostructures includes monoglycerides, glycerate surfactants, glycolipids, phosphatidylethanolamines, and urea derivatives [7]–[15]. Among these amphiphilic surfactants, of particular interest for both basic research and technological applications are monoglycerides: they are biodegradable, biocompatible, and considered as GRAS (generally recognized as safe) materials forming different self-assembled nanostructures. Depending on thermodynamic parameters they self-assemble in aqueous medium to form various nanostructures: reverse isotropic micellar (L_2_), lamellar (L_α_), inverted type hexagonal (H_2_), and cubic (V_2_) liquid crystalline phases [7]–[9].

Especially, the unique properties of the non-lamellar phases (the H_2_ and V_2_ phases) have attracted much interest as *soft* nanoobjects-of-choice in food and pharmaceutical applications, essentially because of the increasing evidences for their biological importance inside living cells [16]–[19]. Their important role in biological processes such as membrane fusion, fat digestion, and control of protein functions and interactions is widely discussed [16]–[22].

In addition, cubosomes (aqueous dispersions of V_2_) and hexosomes (aqueous dispersions of H_2_) have gained considerable interest owing to their unique physicochemical properties [23]–[25]. The first examples on the formation of these colloidal aqueous dispersions were reported by Larsson and his co-workers [26]–[30]. These nanostructured aqueous dispersions have also attracted remarkable attention owing to their potential applications in solubilizing food and active biomolecules with different physicochemical properties (hydrophilic, hydrophobic and amphiphilic active components) [23]–[25], [31], [32]. For instance, different studies showed that these nanostructure aqueous dispersions are promising for loading both hydrophobic and hydrophilic drugs [33]–[37].

Different groups have studied the formation and the characterization of the inner periodicity of *new* nanostructured dispersions based on different surfactant-like lipids such as MO [28], [29], MLO [9], phytantriol (PHYT) [38], glycerate surfactants [39], glycolipids [40], and phospholipids mixed with small amounts of PEGylated MO [41]. Recent investigations were predominantly focused on the effect of temperature [9], [42]–[46] and solubilizing guest molecules (such as hydrophobic additives and proteins) on the confined nanostructures of monoglycerides-based cubosomes [32], [42], [43], [45], [46].

**Figure 1:**
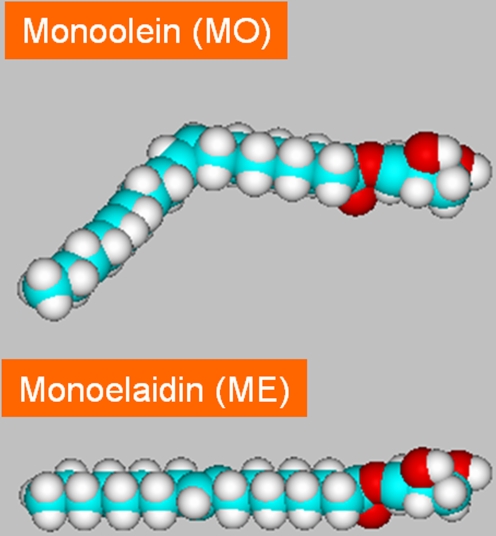
The molecular structure of the lipids: MO and ME.

MO and ME are neutral monoacylglycerols having the same molecular weight, but with different molecular shape. ME has *trans* double-bond located at the 9,10 position in its straight acyl chain (C18:1t9). However, its counterpart MO has a different configuration (*cis* double-bond in the carbon atom backbone, C18:1c9), which causes a “kink” in the middle of the molecule and reduces its effective length (**Figure 1**). The ME/water self-assembled system undergoes lamellar to non-lamellar transitions and hence is well suited to serve as a model system mimicking the different steps of membrane fusion that occur in the biological cells, e.g. in endo- and exocytosis [12], [47]–[50]. In this context, one interesting feature of the thermotropic behavior of the non-dispersed ME/water system is the unique direct transformation under full hydration conditions from L_α_ to V_2_. This behavior is different from the observed temperature dependence of the majority of fully hydrated monoglycerides-based systems [8], [9], [11], [51]. For instance, the order of the phase transitions in MO/water and MLO/water systems under full hydration conditions is V_2_ (at ambient temperatures) via H_2_ to fluid isotropic inverse micellar solution (L_2_, at high temperatures) [8], [9]. In a recent investigation, the analogue transformation from cubosomes via hexosomes to ELP (emulsified L_2_ phase) was reported for the MLO-based aqueous dispersions during heating cycle from 25 to 94°C [9]. The transition was also reversible during heating-cooling cycles and it was identical to that observed in non-dispersed phases [9].

Also recently, Conn et al. [49] reported on the dynamics of the phase transition of the fluid fully hydrated L_α_ to cubic assemblies with *Pn3m* (diamond type, C_D_) and *Im3m* (primitive type, C_P_) symmetries in the binary non-dispersed ME/water system by carrying out both temperature- and pressure-jump investigations. They proposed that the fluid L_α_ phase consists of closely packed onion-like vesicles. They hypothesized that the onion vesicle confines a highly swollen cubic phase that acts as a seed for the formation of an ordered Pn3m phase via the formation of stalks and then fusion pores. For general models on membranes fusion intermediates, the interested reader is directed to the following works [21], [22], [52], [53].

In the present study, we report on the preparation of ME aqueous dispersion and the characterization of its colloidal nanostructured particles. The stabilization of this dispersion was done by adding the polymeric stabilizer Pluronic F127 during the dispersing procedure. Herein, our major goal is to focus on answering the following main question: is it possible to induce a direct transition of vesicles to cubosomes by heating a ME-based aqueous dispersion? For this purpose, we also compare the internal nanostructure of ME-based aqueous dispersion to that of the fully hydrated bulk non-dispersed ME phase.

## Results and Discussion

### 1. Tuning the Internal Nanostructure Curvature from L_α_ to V_2_

**Figure 2:**
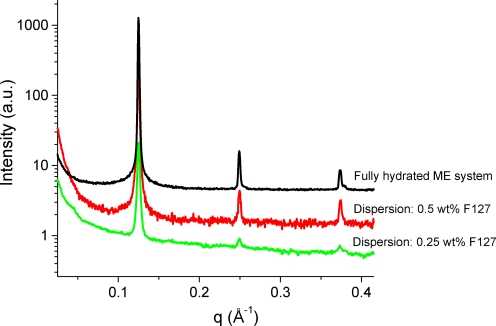
SAXS patterns at 25°C comparing different sample preparations. From bottom to top: scattering curve of ME-based aqueous dispersions consisting of 95 wt% buffer and of a 5 wt% ME/F127 mixture with 0.25 wt% F125 (green), and with 0.5 wt% F125 (red) as well as fully hydrated ME multilamellar vesicles (black). The scattering curves have been normalized and are shifted by an arbitrary constant for better visibility.

**Figure 2** presents the SAXS scattering curves for two different ME-based aqueous dispersions and a bulk non-dispersed fully hydrated ME system at 25°C. The dispersions were formed at two different F127 concentrations: 0.25 and 0.5 wt%, respectively. At this temperature, it is worthy noting that the nanostructure of the fully hydrated ME non-dispersed phase is well preserved (the fluid L_α_ phase of the liposomal dispersion is identical to that of the non-dispersed phase). The peaks of the dispersions are just lowered in the scattered intensity, which can be attributed to the effect of dilution or possibly also the formation of small aggregates of ME with F127 in co-existence with the dispersed liposomal particles. In all investigated samples, the results show three peaks in the characteristic ratio for a lamellar (L_α_) phase with a *d*-spacing of 50.4 Å. This implies that the polymeric stabilizer F127 does not affect significantly the L_α_ phase.

**Figure 3:**
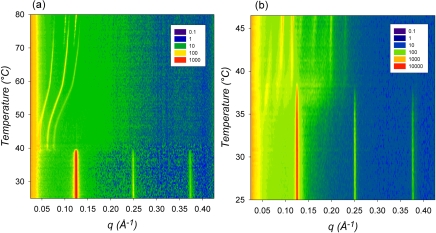
Contour X-ray diffraction plots covering the phase regime of the L_α_, Im3m and Pn3m phases and displaying all the strong peaks as a function of *q* and temperature. (A) In a temperature scan from 25 to 80°C with a rate of 1°C/min, the ME aqueous dispersion underwent the internal L_α_ *via* Im3m to Pn3m structural transition. Every minute 3 X-ray patterns were recorded, i.e. the temperature resolution was 0.33°C. The exposure time was 5 sec (wait interval 15 sec). (B) The fully hydrated ME/water system displays the same three phases in a narrower temperature range, i.e. within 10°C only. Hence, a lower scan rate of 0.5°C/min was applied. It should be noted that at 1°C/min the formation of the Im3m phase is only scarcely visible (data not shown). X-ray patterns were recorded every half a minute, i.e. the temperature resolution was 0.25°C. The exposure time for each X-ray scattering curve was 10 sec (wait interval 20 sec). The color scale of the diffraction intensity is mentioned in the insets.

As mentioned in the introduction, heating the non-dispersed fully hydrated bulk ME aqueous system induces a structural transition in the order of L_α_→V_2_ (mixture of Im3m and an intermediate Pn3m phase)→V_2_ (Im3m)→V_2_ (Pn3m). In this context, we first set out to verify, if an analogous temperature-dependent structural order could efficiently be induced in the interior of ME-based dispersions. In other words, our main interest in this study is to check, if a direct transformation of liposomes to cubosomes by heating the dispersions is possible. **Figure 3a** shows an example of the temperature induced internal structural phase transitions observed in ME-based dispersion consisting of 4.5 wt% ME, 0.50 wt% F127, and 95 wt% PBS buffer in a temperature range of 25–80°C. Our results clearly indicate that temperature increase induces an internal structural transition from L_α_ to the Im3m and then to the Pn3m cubic phase. It is clear that especially for the cubic phases region only few peaks were detectable, but the indexing was consistent with other results presented later in this section on the fully hydrated bulk ME-water system and also with previous reported observations [12]. Czeslik et al. [12] suggested that the relative high membrane elasticity could be the reason for the observation of large size of the cubic lattices in the fully hydrated ME-based systems. This in turn would explain the short-range order in the highly swollen cubic phases and be reasonable for detecting only few orders of diffractions. As a control experiment, the same investigation was carried out on the non-dispersed fully hydrated ME/water system (**Figure 3b**), but a reduced temperature range was sufficient to cover all three phase regimes (25.0–46.5°C). While the disappearance of the L_α_ phase is observed in both cases at approximately 38°C, the transition from the Im3m to the Pn3m phase in the fully hydrated non-dispersed sample takes place already at approximately 40°C and not as seen in the dispersion at approximately 75°C. In this context, the influence of F127 on ME dispersion is in agreement with similar investigations that were done on the aqueous dispersions of other monoglycerides such as MO- and MLO/diglycerol monooleate (DGMO)-based systems. It was found that the internal cubic phase transforms from Pn3m to a symmetry of Im3m as a result of the polymer partition into the cubic phase [29], [42], [54]. This is clearly seen also in our experiment: the temperature range of the Im3m phase is almost 7-fold enlarged. In contrast, a different behavior was recently observed when the oil-free and oil-loaded monoglycerides systems based on either MLO or PHYT were dispersed in aqueous medium in the presence of F127 [9], [43], [45]. In those investigations, it was reported that F127 is an efficient stabilizer that covers mainly the outer surface of the dispersed particles and thus the internal nanostructure of these dispersions was retained at all investigated temperatures [9], [43].

Landh [55] studied the phase diagram of the ternary MO/water/F127 system in detail. He found that the cubic phase extends through a wide range of water (up to 67 wt%) and a maximum polymer content of 20 wt%. Larsson also reproduced in a simplified manner the obtained partial phase diagram [27]. They show that F127 is incorporated into the monophasic MO-based cubic phase region, which induces transitions between different cubic symmetries and significantly enhances the water solubilization capacity in these self-assembled systems. A different phase behavior was reported for the ternary bulk MLO/water/F127 system [9], [42], in which it is difficult to insert the stabilizer's molecules in the monophasic cubic phase region. Therefore, already a small amount of F127 leads to biphasic samples (Pn3m cubic phase coexists with polymer-rich aqueous medium). However, a possible route to induce the insertion of F127 molecules in these phases and to favor the formation of relatively larger hydrophilic channels was achieved by replacing part of MLO by DGMO [42]. As judged from the previous investigations, it is not surprising that the impact of F127 on MLO dispersions significantly differs than that of MO dispersions. In the former dispersions, F127 is an efficient stabilizer and its addition does not affect the symmetry of the dispersed phases. It additionally leads to the formation of internal nanostructure containing identical amount of solubilized water to that solubilized in the bulk non-dispersed sample [9], [42]. However, MO internal nanostructure significantly swells water in the presence of the polymeric stabilizer as a result of the stabilizer's incorporation into the ordered interior of the dispersed cubosome particles. In a previous study [42], it was proposed that this difference might simply be related to the size of the hydrophilic channels in these systems. In the case of MLO/water system, the hydrophilic channels are smaller than those in the MLO/DGMO/water or MO/water systems and therefore the polymer cannot be incorporated as easily in the former case. In our study, a similar explanation can be proposed taking into consideration the fact that F127 does not affect the L_α_ phase of the ME dispersion at 25°C (**Figure 2**), which suggests that the water spacing between neighboring membranes is too narrow and does not leave enough space for the PEG headgroups of the polymeric stabilizer. However, further investigations are required to fully understand the phase behavior of the ternary ME/water/F127 system.

Furthermore, there is another difference between the ME dispersion and the fully hydrated bulk sample. In the former, the temperature induced L_α_ to Im3m transition is not always direct as shown in **Figure 3a**, but it may occur *via* the formation of an intermediate Pn3m phase as described for the bulk phase in [12]. It should be pointed out that this intermediate phase is metastable [12], and it was observed also in our study, when applying higher scan-rates on the fully hydrated phase (data not shown). In other studies [47], [48], it was also reported that the transition of L_α_ to Im3m is not sharp, but it evolves non-reproducible formation of an intermediate phase [48]. Therefore, before data acquisition of the kinetic structural transitions by temperature- and pressure-jump experiments, the fully hydrated system were thermally cycled several times between −20 and 70°C in order to reduce the number of oversized particles [49]. It was argued that temperature cycling would help to homogenize the ME-liposome size distribution and decrease its overall mean size, and thus guarantee better reproducibility.

The lipid molecular structure has a very significant effect on the temperature-composition phase behavior [7]–[9], [11]. Compare for example the influence of replacing ME (a monoglyceride containing a *trans*-monounsaturated acyl chain) at ambient temperatures and under full hydration conditions by its *cis*-monounsaturated counterpart MO (**Figure 1**). Although both lipids have the same molecular weight, their behavior is significantly different: the ME/water system displays planar bilayers [47]–[49], while its analogous MO forms in excess water saddle-like curved bilayers (bicontinuous cubic Pn3m-phase with negative membrane curvature) [8], [26]. A simple approach to explain theses differences is given by the critical packing parameter (CPP), which is defined as
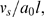
(1)where *v_s_* is the hydrophobic chain volume, *a_0_* is the headgroup area, and *l* is the hydrophobic chain length [56]. In this respect, the time-averaged molecular shape of ME in the fluid phase can be considered rod-like, thus it has a CPP value of approximately 1 and favors the formation of planar bilayers at room temperature. It is worthy noting that the values of *a_0_* and as well as *v_s_* above the chain melting transition are nearly equal for ME and MO [57], but in contrary MO has due to its *cis*-bond in the chain a reduced chain lengths, *l*, and hence its CPP is greater than unity. Thus, the time-averaged truncated conical shape of the MO-molecules can be considered as the driving force to form self-assembled nanostructures with negative spontaneous monolayer curvatures.

It is important mentioning that at room temperature F127 is not an effective stabilizer for the ME dispersion, which is significantly less stable than its counterpart MO-based aqueous dispersion (stable for months). Few days after preparation, they lose their stability. In an attempt to improve the kinetic stability of these dispersions, we decided to prepare them with higher stabilizer's concentrations. However, we found that with an increase of stabilizer's concentration above 0.5 wt%, there is even a significant reduction in the stability of the formed gel-like dispersion. A possible explanation for this difference between ME and MO dispersions might simply be related to the molecular geometry of the investigated lipid and the accessibility of the polymer molecules on the outer surface of the dispersed particles. In ME-based dispersions, the polymeric stabilizer tends most probably to maintain in the surrounding aqueous medium without being significantly located at the outer surface of the kinetically stabilized ME particles. In contrast, the mechanism of the steric stabilization in the MO case is strongly enhanced by the presence of a considerable amount of F127 molecules at the external surface of the dispersed particles: the polymer mainly adheres to the surface of the dispersed particles to provide stable nanostructured aqueous dispersions [26], [28], [29]. It should be pointed out that different reports confirmed the hypothesis that the behavior of *trans* fatty acids closely resembles saturated fatty chains rather than those of *cis* fatty acids [58], [59]. The *cis* double bond inhibits close packing of the acyl chains, whereas the straighter trans chains induce a tighter lipid packing in the interfacial polar-apolar area [59]. Thus, we assume that this alteration in the lipid packing most likely has an effect on the adsorption rate of F127 on the surface of the dispersed particles. While F127 easily adheres to membranes with high fluidity, its impact on tighter packed lipid membranes is less pronounced. Thus, the addition of F127 probably does not efficiently adsorb at the dispersed ME particles, and hence a high amount of the stabilizer remains in the excess of water. In this case, it has been shown that F127 forms self-assembled normal spherical micelles in the aqueous medium [60]–[62]. They in turn can induce a depletion interaction between the particles leading to a flocculation and an increase in the viscosity of the aqueous phase (formation of gel-like dispersions). In this respect, our results suggest that the ME particle-particle interactions become even more prominent when increasing F127 concentration. The influence of adding non-adsorbing polymers and polysaccharides on the depletion interaction was discussed in various studies in literature. Few examples are given in [63]–[65].

**Figure 4:**
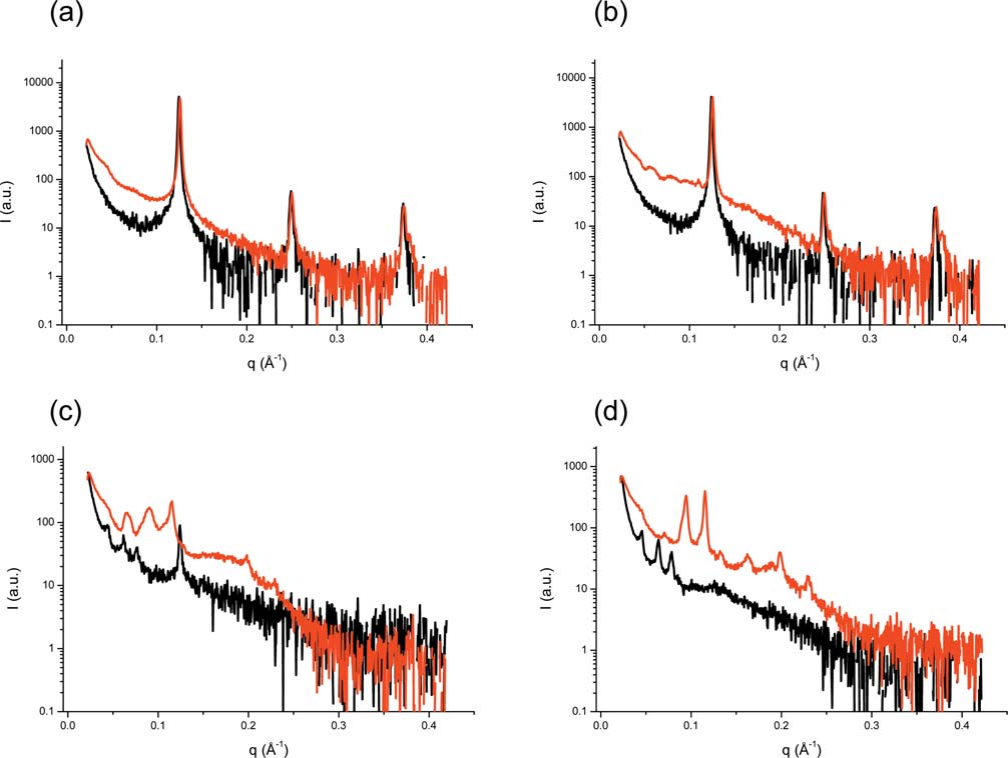
Comparison of the scattering curves of a ME-based dispersion and a fully hydrated bulk sample. At four different temperatures, namely, at (A) 25, (B) 35, (C) 40, and (D) 45°C a ME-based emulsified system (black lines) is compared with a non-dispersed fully hydrated bulk sample (red lines). The intensities were normalized at high q. The ME dispersion were prepared with a F127 concentration of 0.5 wt%.

To gain further insight into the structural transitions, representative scattering patterns of the dispersed ME/water phase as well as from the fully hydrated ME phase at four different temperatures are plotted in **Figure 4**. The SAXS analysis at both states provides clues to understanding the influence of the polymeric stabilizer on the internal nanostructure of this aqueous dispersion. Our results clearly indicate that the internal nanostructure of the ME-based dispersion is different from that of the fully hydrated ME system, when both systems were heated above 38°C. This structural disagreement suggests that a considerable amount of F127 remains in the aqueous medium and hence is also significantly present in the interior water regions of the dispersed ME particles. Following this train of thought an increase in temperature, which increases the fluidity of the ME-bilayers, not only induces the L_α_ to cubic phase transition above a certain threshold of chain disorder, but also allows a significant incorporation of F127 into the polar interface of the ME-membranes. For instance, at 40°C the lattice parameter values for the dispersed and the bulk samples are 205 and 134 Å, respectively. The formation of highly swollen Im3m in the dispersion is believed as discussed above to arise from the incorporation of F127 in the internal nanostructure after the transition from the fluid L_α_ phase. This is clearly different to MO stabilized particles, in which F127 efficiently plays its role as stabilizer: it mainly covers the outer surface of the dispersed particles, but still a certain amount is incorporated into the internal nanostructure, however, its incorporation should be less pronounced then in the ME-based cubosomes. Thus, the internal structural transition from Pn3m to Im3m depends on the polymeric stabilizer's concentration [29], [54]. In summary, our results suggest that the transition of L_α_ (**Figures 4a & 4b**) to Im3m (**Figures 4c & 4d**) as a result of membrane fusion processes [21], [52], [53] takes place through the interplay of the following steps: (1) enhancement of the lipid chain fluidity, (2) water uptake, and (3) penetration of F127 into the membrane surfaces.

Interestingly, the L_α_-Im3m structural transition in the dispersed and the bulk samples involves a extreme shift of the positions of the observed Im3m peaks to lower *q* values, which signifies an enlargement of the nanostructure (Im3m phase) as a result of water uptake. This water swelling behavior is different from most reported studies on the influence of varying temperature on the structure of fully hydrated monoglycerides-based systems and their nanostructured aqueous dispersions [8], [9], [45]. For instance, the temperature dependence of the lattice spacings for all fully hydrated phases and their aqueous dispersions in both dispersed and non-dispersed MO/water and MLO/water systems can be ascribed solely to variations in the CPP value, i.e. a reduction in the value of *a_0_* (the headgroup area) due to dehydration and a simultaneous enhancement of the value of *v_s_* (the hydrophobic chain volume) explains well the changes of *a(T)* [8], [9], [43], [66].

**Figure 5:**
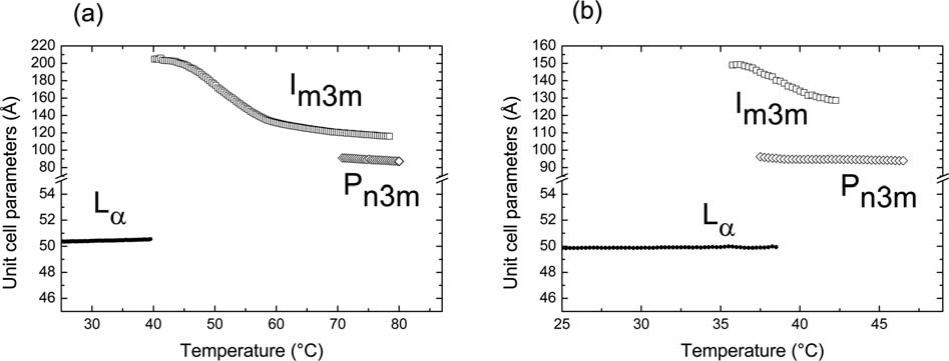
Variation in the unit cell parameters as a function of temperature for the L_α_, Im3m and Pn3m phase. The lattice parameters of the different phases formed in the ME-based aqueous dispersion (a) are compared to its fully hydrated system (b). The data are extracted from the experiments displayed in Figure 3.

The temperature-dependent evolution of the derived lattice parameters of the ME/water systems is illustrated in **Figure 5** for the two different states (dispersed *vs.* bulk samples). There is almost no significant change in the lattice parameter of the L_α_ and the Pn3m phases with increasing temperature. This is in agreement to the experimental data on ME/water bulk samples [12]. It is worthy noting though, that Templer and colleagues [49] observed under condition of full hydration during the time course of temperature-jump experiments a shrinkage in the lattice parameter of the L_α_ phase and the existence of a highly swollen intermediate cubic phase.

However, the lattice parameter, *a(T)*, of the Im3m phase significantly reduces with increasing temperature. The *a(T)* decreases with a rate approximately of −5.1 Å/°C for the dispersion in a temperature range of 45–68°C, whereas the decrease is approximately −3.6 Å/°C for the fully hydrated sample in a temperature range of 37–42°C, which is in agreement with the value reported in [12]. This reduction is attributed to the temperature-induced headgroup's dehydration. Additionally the Im3m phase expels water to the surrounding aqueous medium with increasing temperature, while there is no significant change in the Pn3m structure. In this case, the results indicate that part of the polymeric stabilizer located in the internal nanostructure is expelled together with water when the dispersion is heated above the L_α_-Im3m transition temperature.

**Figure 6:**
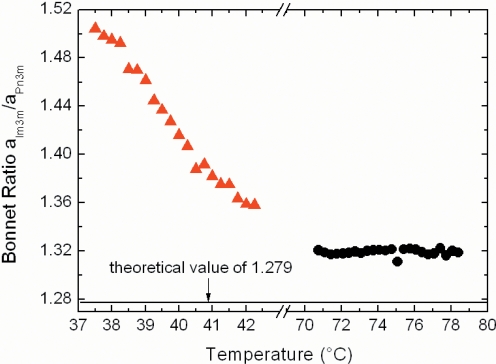
Variation in the Bonnet relation as a function of temperature. The Bonnet relation is expressed in the ratio of the cubic unit-cell parameters of the Im3m and Pn3m. The deviation from the theoretical value of 1.279 (dash-dotted line) is relatively small in the ME-based aqueous dispersion (black circle), while strong deviations are found for its fully hydrated system (orange triangle). The data are extracted from the experiments displayed in Figure 3 (compare also Figure 5).

Next to the comparison of the lattice parameters of the ME-bulk and dispersion samples, also the coexistence region of the two cubic aspects shows up the different hydration properties (**Figures 3** and **5**). The bicontinuous phases Im3m and Pn3m contain both minimal surfaces (primitive and diamond minimal surfaces), which can be identified with the mid-plane of the membrane bilayers [17], [26]. Under equilibrium conditions, the averaged Gaussian curvatures of two coexisting bicontinuous cubic phases should be the same. Therefore, it can be easily shown as a consequence that the ratio of the unit cell parameters of the involved Im3m and Pn3m phases should take the theoretical *a*_Im3m_/*a*_Pn3m_ value of 1.279 (this ratio is known as Bonnet relation) [17], [67], [68]. As shown in **Figure 6** for the ME dispersion, the Bonnet ratios lie in the range of 1.31–1.32 in a temperature interval of 70.7–78.4°C. These values are in good agreement with the theoretical value, and with the reported ratio of 1.33 for the glycolipid-dispersed cubosome particles with confined Pn3m-Im3m coexisting phases [40]. Also the measured values for MLO/diglycerol monooleate (DGMO)-based aqueous dispersions (*a*-axis ratios of 1.30–1.34) are consistent with the Bonnet relation [42]. Further examples on lipid/water systems that are consistent with the Bonnet ratio are given in the review of Larsson and Tiberg [67]. It can be seen from **Figure 6** that the *a*_Im3m_/*a*_Pn3m_ ratio for the bulk ME sample in a temperature range of 37.5–42.2°C is differently behaving than the dispersion. At 37.5°C, the obtained *a*-axis ratio of 1.50 is clearly not consistent with alike Gaussian surface curvatures. Upon heating the fully hydrated lamellar phase transforms first into the Im3m phase and then the Pn3m phase evolves. As can be understood from **Figure 5b**, the initially high *a*-axis ratio is most probably caused by the anomalously swollen Im3m phase. However, this significant deviation from the theoretical ratio decreases with increasing temperature. Further increase of temperature induces a great reduction in the *a*_Im3m_ value (a decrease in solubilized water content); whereas there is no significant change in the *a*_Pn3m_ value (the same amount of solubilized water). Therefore, at 42.2°C the Bonnet ratio was found to be approximately 1.36 and in reasonable agreement with the theoretical ratio for the Pn3m-Im3m transition.

### 2. Cryo-TEM Observations of the ME-based Aqueous Dispersion

In order to gain further insight into the nanostructures the ME-based aqueous dispersion and to directly visualize the morphology of the dispersed phase, Cryo-TEM experiments have been performed. This technique is widely used to verify the SAXS results and to characterize the dispersed particles of liquid crystalline phases and microemulsions [9], [43], [46], [69]–[71]. Previous studies on the Cryo-TEM imaging of MO-based cubosome and hexosome particles revealed important information on the shape of the dispersed particles and their confined nanostructures [26], [28], [29], [69]. The present study also allows gaining further information on the main differences in the morphology of the dispersed particles of the *cis*-monounsaturated monoglyceride MO as substituted by its *trans* homologous lipid ME.

**Figure 7:**
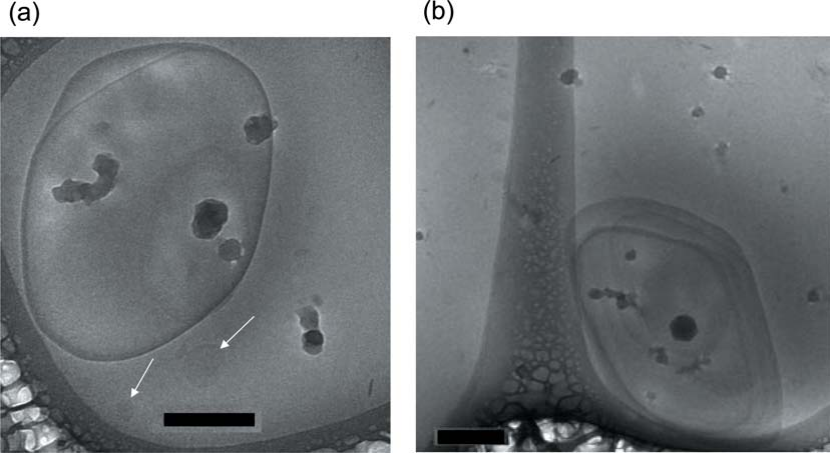
Cryo-TEM images of ME-based aqueous dispersion containing 1.5 wt% lipid, and 0.035 wt% F127 after vitrification from 25°C. (A) A stabilized multilamellar vesicles with a size of about 500–600 nm is highlighted in dark cyan. Rarely free membranes are visible (white arrows). We note, that the dark spots in these images and in the next figures indicate the formation of ice crystals on the microscope grids. (B) Another example of closely packed onion vesicles consisting of concentric bilayers. The image also indicates that the morphology of most dispersed multilamellar vesicles seems to be angular or most likely compressed. Scale bar is 200 nm in the images.

**Figure 7** shows two images with higher magnification of ME-based aqueous dispersion after vitrification from 25°C. The image presented in **Figure 7a** indicates most likely the formation of very tightly multilamellar vesicles with few hundredths of nanometer in diameter. In this case, it is difficult to observe the individual bilayers as a result of the closely packed aggregates. This fits to the interpretation is given in [49], in which it was argued that ME forms in water closely packed onion vesicles consisting of concentric bilayers. However, it is worthy noting that in panel A also the formation of free bilayers in some small regions is seen (see the arrows). Moreover, the shape of the stabilized vesicles is in general not perfectly round, but often shows some angularity, most probably due to vesicle compression (**Figure 7b**).

It is important to note that the dispersions display a broad particle size distribution. Very large micron-sized ME objects with a diameter of a few microns were observed at small magnification (data not shown). Even larger particles were often attached to the grids. These thick areas were impenetrable to the electron beam, and could not be studied. They had also a further detrimental effect by causing an excessive formation of ice crystals also on adjoining thinner film areas, making the quality of the micrographs low. Therefore, it is difficult to obtain details of the internal structure. We also recall that Conn et al. [49] reported on a high particle size distribution in the fully hydrated binary ME/water system.

**Figure 8:**
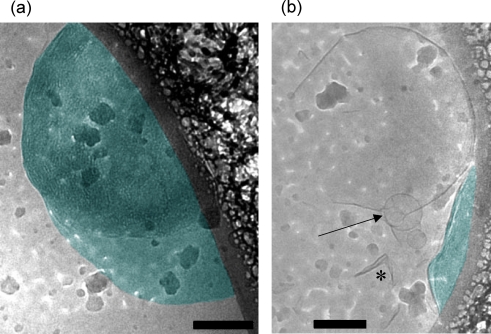
Cryo-TEM images of ME-based aqueous dispersion containing 1.5 wt% lipid, and 0.035 wt% F127 after vitrification from 65°C. The dispersion was ultrasonicated and extruded through 200 nm filters before the investigation. (A) A stabilized ME-based particle with inner cubic structure highlighted in dark cyan. The inner nano-tubular network is clearly visible. Judging from the X-ray data the inner lattice should be of the symmetry Im3m. The overall shape, however is not well defined as compared to other cubosomes known from literature [9], [29], [73]. (B) An adhered cubosome of lengthy shape is seen (dark cyan). In other areas relatively large open vesicles with a cluster of small vesicles is visible (marked by an arrow).

To further explore the formation of the V_2_ phase at higher temperatures as indicated by SAXS analysis, we also studied the morphology of the ME dispersion at 65°C. Most images are similar to those presented in **Figures 8a and 8b** and indicate the formation of particles with internal cubic structure (colored in dark cyan). Although the obtained images from ME/water dispersions are not very clear, an ordered structure is visible inside the formed cubosomes. Their symmetry though remains obscured, and we can only indicate from the given SAXS data that the interior consists most likely of the Im3m phase. The formed cubosomes have great tendency to adhere to the polymer film. This enhanced interaction leads to the formation of flattened cubosomes adsorbed into the edge of the polymer film and covers a large region (few hundreds of nanometers). It is worthy noting that some MO cubosome objects attaching the polymeric edge was also already observed in previous Cryo-TEM investigations [29], [70].

As judged from **Figure 8**, the findings indicate that replacing MO by the trans analogous ME induces unusual cubosome morphology that is significantly different from the typical well-shaped cubosome and hexosome particles, when monoglycerdies such as MO, MLO, and PHYT were dispersed in water [9], [28], [29], [45], [69], [72], [73]. In general, the Cryo-TEM observations indicate the formation of high fraction of sterically well-stabilized cubic- or rounded-shaped dispersed cubosome or hexosome particles with a distinctive ordered interior [9], [28], [29], [69], [72]–[75]. However, the ME dispersion does not display such well-shaped peculiar particles (globular or faceted objects), but rather large objects attached to the polymer film. Last to be pointed out, a number of crumbled bilayer fragments have been observed in different views (as seen in Figure 8b, *), and we also noted in few images the formation of open vesicles as marked by an arrow in this figure. Theses features are usually not observed-at least not in this frequency-for MO cubosomal dispersions, and hence might be due to particular transition mechanism when passing from lamellar to non-lamellar structures. However, further examinations will be necessary to clarify this point and clearly goes beyond the scope of the underlying study.

In conclusion, we have investigated in the present work by a combination of SAXS and Cryo-TEM techniques the temperature-induced structural transitions of ME-based aqueous dispersion as compared to its fully hydrated ME phase. Our ultimate goal was to experimentally demonstrate the direct transition from vesicles to cubosomes by heating the dispersions. This included understanding the mechanism and properties of the involved lamellar to non-lamellar transition under the influence of a stabilizing agent (F127). Moreover, the vesicle to cubosome transition is of special interest, because it gives direct information also on the influence of stabilizers in membrane fusion processes.

The formation of these kinetically stabilized dispersions was achieved in the presence of the polymeric stabilizer F127. They were significantly less stable than those based on its congener MO. Our SAXS findings suggest that the stabilizer F127 is significantly affecting the internal nanostructure as the dispersions are heated above 25°C. We found that the direct L_α_-V_2_ transition in the dispersion does not reveal the same mechanism as that of its fully hydrated bulk system. Notably, the polymer incorporation into the internal nanostructure leads to the formation of highly swollen Im3m phase as the membrane fusion processes take place.

Our Cryo-TEM observations provided further insight into the morphology of the obtained vesicles after vitrification of the dispersion from 25°C. Additionally, information concerning the internal nanostructure of the dispersed cubosome particles was obtained as the vitrification performed at higher temperature (65°C). Actually, the obtained images were not very clear and are significantly different from those published for the analogous MO dispersions. At 25°C, the formation of most likely closely packed onion-like vesicles with broad vesicle size distribution has been seen. Interestingly, the images at 65°C show flattened cubosomes with internal nanostructure that adsorb to the edge of the polymer film without displaying any particular shape. Therefore, they are different from the dispersed well-shaped cubosome and hexosome particles formed in MO dispersions.

## Materials and Methods

### Materials

Monoelaidin (1-mono[*trans*-9-octadenoyl]-rac-glycerol, ME, purity: 99%) was obtained from Larodan Fine Chemical (Malmö, Sweden). Chloroform (CHCl_3_, purity: > 99%) was supplied by Carl Roth GmbH (Karlsruhe, Germany). The used buffer was PBS (phosphate buffered saline contains 20 mM NaPi and 130 mM NaCl, pH 7.4). The stabilizer Pluronic F127 (PEO_99_-PPO_67_-PEO_99_) was a gift from BASF Corporation (Mount Olive, New Jersey, USA). All ingredients were used without further purification.

### Preparation of ME/Water Bulk Non-Dispersed Samples

ME was dissolved in chloroform. This solvent was then evaporated using a gentle stream of nitrogen, followed by drying under vacuum for at least 12 hours in order to remove completely the residual organic solvent. The dry lipid film was hydrated by adding the PBS buffer and carrying out at least 5 freeze-thaw cycles between liquid nitrogen and room temperature and then homogenizing several times during the thawing steps by vigorous vortexing. The fully hydrated sample coexists with excess water was formed with a fixed total lipid concentration of 30 wt%. The prepared sample was incubated at room temperature for two to three weeks before carrying out SAXS measurements.

### Formulation of Two ME-Based Aqueous Dispersions

Appropriate amounts of the PBS buffer containing a given concentration of F127 were added to the ME dry lipid film. Then the samples were ultrasonicated for 10 min resulting in a milky dispersion. The typical composition of the prepared two dispersions was 95 wt% buffer and 5 wt% of a ME/F127 mixture, whereby the concentration of the F127 was 0.5, and 0.25 wt%, respectively. Ultrasonication was carried out using the high intensity ultrasonic processor VCX130 (SY-LAB GmbH, Neupurkersdorf, Austria), at 60% of the maximum power (the maximum power is 130 kW), with 0.5 s pulses interrupted by 0.5 s breaks. The resulting milky aqueous dispersions were stable at 25°C only for few days.

### Time-Resolved Synchrotron X-Ray Scattering Measurements

X-ray scattering patterns were recorded at the Austrian SAXS beamline [76] (camera length 125 cm) at the synchrotron light source ELETTRA (Trieste, Italy) using a one-dimensional position sensitive detector (Gabriel type), which covered the *q*-range (*q = *4π *sinθ/λ*, where *λ* is the wavelength and *2θ* is the scattering angle) of interest from about 2π/640 to 2π/13 Å^−1^ at an X-ray energy of 8 keV. Silver behenate (CH_3_-(CH_2_)_20_-COOAg with a *d* spacing value of 58.38 Å) was used as a standard to calibrate the angular scale of the measured intensity. The lipid-samples were sealed in a thin-walled quartz capillary (Anton-Paar, Graz, Austria) and thermostated with a programmable water bath (stability ±0.1°C, Unistat CC, Huber, Offenburg, Germany). Before carrying out the continuous temperature scan experiments, static measurements were done at 25°C with an exposure time of about 120 sec. The temperature scans were performed at scan rates of 0.2 to 1°C/min. Typically 2–3 X-ray patterns were recorded per minute with a constant exposure time of about 10 sec. Between each exposure, a small solenoid driven shutter blocked the direct X-ray beam in order to minimize the total radiation dosage on the sample.

### X-ray data-analysis

In the time resolved X-ray scattering experiments, the lattice spacings of the L_α_, the cubic Im3m, and the Pn3m phases were derived from the SAXS diffraction pattern by standard procedures as described in [77]. In short, after the raw data had been corrected for detector efficiency and the background scattering both from water and the sample cell had been subtracted, all Bragg peaks were fitted by Lorentzian distributions. We note that in each respective phase regime only the strongest reflections were considered. The fittings were carried out with home written procedures running under IDL 5.2 (Research Systems, Inc., USA).

### Cryogenic Transmission Electron Microscopy (Cryo-TEM)

The sample was placed in the controlled environment of the vitrification chamber at the chosen temperature where the relative humidity was kept close to saturation to prevent water evaporation from the sample. A 5 µL drop of the aqueous solution was placed on carbon-coated holey film supported by a TEM copper grid. Excess liquid was removed by careful blotting with absorbent filter paper leaving thin biconcave liquid films spanning the holes of the polymer support. The liquid film was then about 10 nm thick at the thinnest parts and 300 nm or more at the edge. The sample was then rapidly plunged into liquid ethane and cooled by liquid nitrogen to its melting temperature to obtain a vitrified film. The vitrified specimen was stored under liquid nitrogen, and transferred in liquid nitrogen into the microscope (Zeiss 902A) operating at zero-loss bright field mode at 80 kV with an underfocus of 1 µm. The working temperature was kept at −165 to −170°C, and the images were digitally collected under low-dose conditions. For further details see Almgren et al. [69].
